# Stability of the PHF10 subunit of PBAF signature module is regulated by phosphorylation: role of β-TrCP

**DOI:** 10.1038/s41598-017-05944-3

**Published:** 2017-07-17

**Authors:** Victor V. Tatarskiy, Yuriy P. Simonov, Dmitrii S. Shcherbinin, Alexander V. Brechalov, Sofia G. Georgieva, Nataliya V. Soshnikova

**Affiliations:** 1 Department of Eukariotic Transcription Factors, Institute of Gene Biology, Russian Academy of Sciences, Vavilov Str. 34/5, Moscow, 119991 Russia; 2 Department of Transcription Factors, Engelhardt Institute of Molecular Biology, Russian Academy of Sciences, Vavilov Str. 32, Moscow, 119991 Russia; 3 Laboratory of Tumor Cell Death, N.N. Blokhin Russian Cancer Research Center, Kashirskoye Shosse 24, Moscow, 115478 Russia; 4 Laboratory of Structure Bioinformatics, Institute of Biomedical Chemistry (IBMC), Pogodinskaya street 10 building 8, Moscow, 119121 Russia

## Abstract

The PBAF chromatin-remodeling complexes are multi-protein machines, regulating expression of genes involved in proliferation and differentiation. PHF10 is a subunit of the PBAF essential for its association with chromatin. Mammalian PHF10 is expressed as four ubiquitous isoforms, which are alternatively incorporated in the complex and differ by their influence on transcription of target genes. PHF10 have different domain structure and two of them (PHF10-S isoforms) lack C-terminal PHD domains, which enables their phosphorylation by CK-1. Here we have found that PBAF subunits have low turnover rate, except for PHF10 which has much lower half-life, and is degraded by β-TrCP. The β-TrCP knockdown stabilizes PBAF core subunits - BRG1 and BAF155 and specific subunits - PHF10, BAF200, BAF180 and BRD7. PHF10 isoforms contain two non-canonical β-TrCP degrons and are degraded by β-TrCP in a phospho-dependent manner. But phosphorylation of PHF10-S degrons by CK-1, contrary to previously described degrons, prevents their degradation. Targeted molecular docking demonstrated that phosphorylated forms of PHF10 bind to β-TrCP with much lower affinity than non-phosphorylated ones, contrary to previously described degrons. This unorthodox mechanism proposes that phosphorylation of β-TrCP degrons by CK-1 could not only degrade a set of proteins, but also stabilize a different set of targets.

## Introduction

The SWI/SNF complexes are large multi-protein machines that remodel the structure of chromatin and interact with a number of co-factors to regulate expression of genes involved in proliferation and differentiation. Two types of complexes, SWI/SNF-a/BAF and SWI/SNF-b/PBAF, have been identified in mammals. Seven core subunits are common for both BAF and PBAF (BRG1 or hBRM, BAF170, BAF155, BAF60, BAF57, BAF53 and BAF47), while BAF180, BAF200, BAF45A/PHF10 and BRD7 subunits are specific to PBAF. The core components of SWI/SNF normally exist only in the complex and not as free subunits, and are very stable when bound together. In an unbound state their half-lives are dramatically shorter than in native equilibrium because the core components are stabilized by each other, especially by BAF155 and BAF 170 that act as scaffolds for the assembly of the rest of the complex and prevent degradation of other components such as BRG1 and BAF57^1, 2^.

The PBAF-specific subunits of the PBAF complex are associated together and form a module which in *Drosophila* has been shown to be essential for interaction with chromatin^3^. Depletion of one of the components of the module decreases the amounts of other subunits of the module^4^. Human PHF10/fly SAYP was found to be a component of PBAF/PBAP-signature module^4–6^ in higher eukaryotes. In mice Phf10 is required for maintenance of neural stem cells during brain development^7^. PHF10 depletion in human cells leads to degradation of other components of the PBAF-signature module, including BAF200, BAF180, and BRD7^5^. In line with this observation the depletion of *Drosophila* PHF10 homologue SAYP destabilizes the specific module of PBAP and dramatically decreases the presence of PBAP on gene promoters^6^.

Previously we have shown that the mammalian PHF10 is represented by four evolutionarily conserved isoforms that differ in the domain structure^5, 8^. The PHF10-P isoforms contain two C-terminal PHD domains which in PHF10-S isoforms are replaced by SUMO1-conjugating motif (PDSM). Both isoforms can be transcribed with the addition of 47 amino acids to the N-terminus (l-isoforms), or without such an addition (s-isoforms). These four variations yield four isoforms designated as Pl, Ps, Sl and Ss. Interestingly, PHF10 isoforms are not tissue specific but are often present in the same cells, although in different ratios, depending on cell type [8]. Different isoforms are alternatively incorporated into the PBAF complex and have different functions. The longest isoform, PHF10-Pl, is needed for proliferation in culture. The PHF10 isoforms undergo an intensive posttranslational phosphorylation, which stabilized their association with the PBAF complex^8^. Nevertheless, other functions of PHF10 phosphorylation are unknown.

The proteins of the PBAF complex are tightly regulated at the level of gene transcription and stability^1^. However, the ubiquitin ligases which participate in degradation of SWI/SNF subunits are largely unknown except for BAF155 (degraded by Wwp2)^9^. Among a variety of E3 ubiquitin ligases that target proteins for degradation the SCF complexes recognize phosphorylated proteins. These complexes contain three constant subunits: Skp1, cullin and Rbx1/Roc1 and one variable F-box protein. β-TrCP is a F-box protein which consists of Skp1-1 binding F-box domain, and a substrate binding WD40 domain. In screening of β-TrCP substrates by Low *et al*.^10^ PHF10 was found to be one of such substrates, with a high confidence score. β-TrCP binds to a consensus phosphodegron motif DpSGXXpS or DpSGXXXpS but can also bind to non-canonical degrons^10–13^. By promoting degradation of crucial cell signaling molecules β-TrCP serves as a major regulator of Wnt, NFκB and p53, thereby modulating cell cycle progression and DNA damage response. Regulation of β-TrCP is achieved by phosphorylation of its substrates and involves protein kinases such as CK-1/2, GSK-3β and others^14, 15^. Casein kinases (CK) phosphorylate β-TrCP substrates β-catenin, mdm2, MTSS1, Cdc25A and many others, enabling binding of β-TrCP to the phosphorylated degron sequences. Thus, CK functions to regulate protein stability.

As PHF10 is an important subunit of PBAF and its low level destabilizes the PBAF-signature module we were interested how the levels of PHF10 and its isoforms are regulated in cells. We measured the half-lives of several PBAF core and specific subunits and found that PHF10 is the most unstable PBAF subunit. In turn, the stability of all four PHF10 isoforms varied dramatically and correlated with phosphorylation patterns. We further demonstrated that PHF10 is subjected to polyubiquitin-dependent proteosome degradation trigged by β-TrCP ubiquitin ligase. The PHF10 isoforms shared two non-canonical β-TrCP degrons; the serine residues within the degrones are phosphorylated by CK-1 in PHF10-S but not in PHF10-P isoforms. Unexpectedly, this phosphorylation decreased the interaction of PHF10-S isoforms with β-TrCP and increased their stability. These results contradict literature data as phosphorylation within the canonical β-TrCP degrons increases their affinity to β-TrCP. The computational analysis of β-TrCP degrons of PHF10 confirmed these findings by showing that non-phosphorylated degrons bind with a high affinity and closer to known structures. Interestingly, the decrease of β-TrCP level in cells stabilized PBAF signature module as well as the whole PBAF complex indicating that β-TrCP is involved in regulation of its stability.

## Results

### PHF10 is the most unstable subunit of PBAF complex

**Figure 1:**
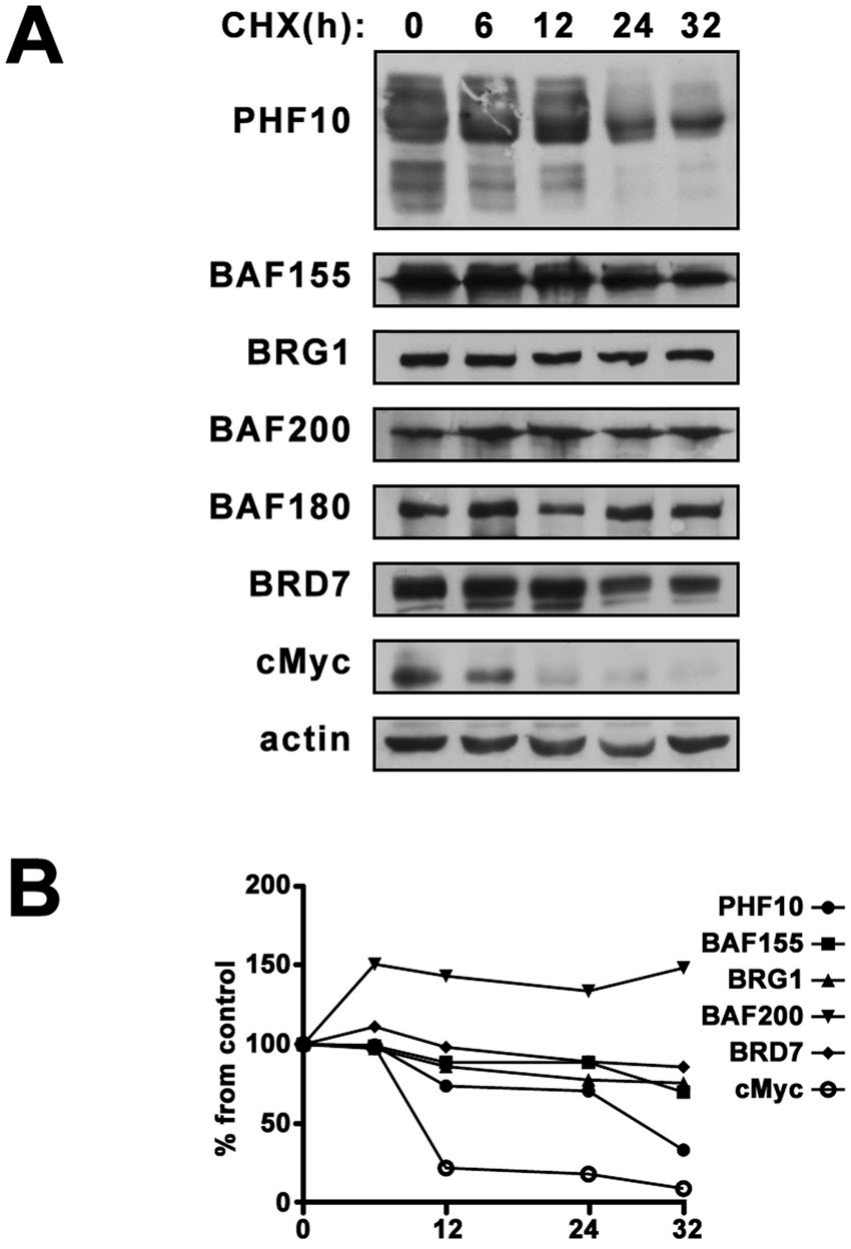
(**A**) The stability of PBAF subunits in human cells. HEK293 were treated with 20 µg/ml cycloheximide (CHX) for the time points indicated above the panels. The equal amounts of lysates of control (0 h) and CHX-treated HEK293 were then analyzed by Western blotting. β-Actin was used as loading control. (**B**) The intensity of bands on Western blot was quantified using ImageJ software by densitometry as described in Methods section. The images in Fig. 1(A) is cropped.

Regulation of protein stability of the core subunits of BAF and PBAF has been extensively studied^1, 16^ but less is known about the stability of the PBAF-specific subunits, in particular, PHF10. In mammalian cells PHF10, similarly to other PBAF subunits, is mainly present in a complex. We analyzed the rate of PHF10 degradation by a cycloheximide (CHX) chase assay in HEK293 cells and compared it with the rate of degradation of core (BAF155 and BRG1) and specific (BAF200, BAF180, BRD7) components of PBAF (Fig. 1). In line with previous reports the core proteins of the complex were very stable. The half-life of BRG1 exceeded 32 h (similar to refs 1 and 17). BAF155 degraded faster with half-lives 24–30 h (similar to ref. 2). It should be noted that half-lives for exogenously expressed SWI/SNF proteins are much lower than for endogenous ones reported above^2^. The half-lives of BAF200 and BAF180 specific subunits exceeded 32 h. The BRD7 degraded more rapidly (half-life ~24 h)^18^. Interestingly, PHF10 was the most unstable subunit of the complex with the half-life of ~12 h.

Such unstable state of PHF10 in the complex can be attributed to its intensive regulation in the cells. Rapid turnover has been demonstrated for highly regulated transcription factors c-Myc and β-catenin^19, 20^. Rapid degradation of c-Myc was also observed in our experiment (see Fig. 1). On the contrary, the half-life of actin exceeded 32 h (Fig. 1).

### PHF10 isoforms are differentially phosphorylated, and X-phosphorylation positively influences the stability of PHF10-S isoforms

**Figure 2:**
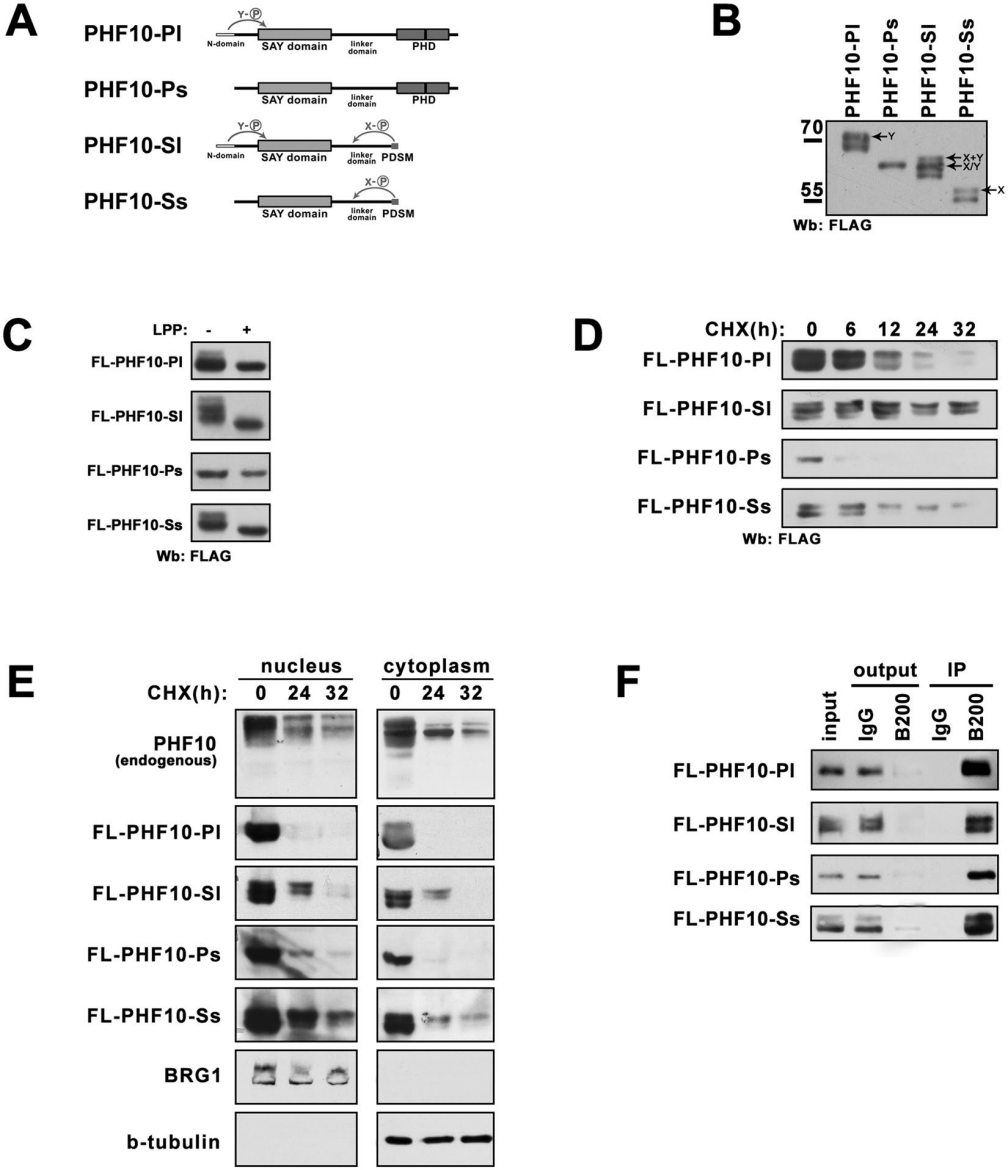
The correlation between stability and phosphorylation of PHF10 isoforms. (**A**) The schematic representation of domain structure and phosphorylation patterns of PHF10 isoforms. N-terminal domain, SAY-domain, linker domain and double PHD or PDSM domains are indicated. N- terminal domain is characteristic for the PHF10-Pl and PHF10-Sl isoforms and determines their Y- phosphorylation pattern. The absence of PHD domains on C- terminus of PHF10-Sl and PHF10-Ss isoforms determines their X-phosphorylation. (**B**) Western blot of FLAG-tagged PHF10 isoforms overexpressed in HEK293. Protein bands corresponding to X- and Y-phosphorylated isoforms are indicated by arrows. (**C**) HEK293 cells transfected with FLAG-PHF10 isoforms. 15 µg of total protein extract were either treated with lambda protein phosphatase (LPP) or left untreatead, and then analyzed by Western blotting (**D**) HEK293 cells transfected with FLAG-PHF10 isoforms were treated with 20 µg/ml CHX for time points indicated above the panels. 15 µg of total protein extract was loaded per well and analyzed by Western blotting. (**E**) Wild type HEK293 cells or HEK293 cells transfected with FLAG-PHF10 isoforms were treated with CHX (20 µg/ml) for different time. After incubation the cells were fractionated into cytoplasmic and nuclear fractions. Protein extracts were analyzed by Western blotting using antibodies against endogenous PHF10 or against FLAG tag. BRG1 and β-tubulin were used as fractionation and loading control. (**F**) The immunoprecipitation (IP) of FLAG-tagged PHF10 from nuclear extract of HEK293 with antibodies against BAF200(B200 in Fig. 2F). The immunoprecipitation demonstrates that all PHF10 is depleted together with BAF200 and thus is associated with PBAF complex. The ½ of precipitates and 1/10 of the Input and Outputs was loaded on the gel. The images in Fig. 2(B,C,D,E, F) are cropped

Next, we studied the stability of different PHF10 isoforms. As shown in our previous study^5, 8^ the PHF10 isoforms differ by the presence or absence of 47 amino acids on their N-termini designated as long (l) or short (s) isoform (Fig. 2A). They also have either two PHD domains or a PDSM (phospho-dependent SUMO1-conjugated motif) domain on their C-terminus designated as P (PHD) or S (sumoylated domain) containing form of PHF10. This yields four PHF10 isoforms designated as PHF-Pl, -Sl, -Ps and -Ss (Fig. 2A). All isoforms share highly conserved SAY domain and a linker domain (Fig. 2A).

To study the rate of degradation PHF10 isoforms were individually expressed as FLAG-tagged proteins in HEK293 cells (Fig. 2B). Most of recombinant PHF10 isoforms migrated as several bands in SDS-PAGE that reflected their extensive phosphorylation^8^. Different phosphorylation pattern of PHF10 isoforms depended on domain structure. The FLAG-tagged Ps isoform which contains PHD domains but lacks 47 N-terminal amino acids migrated as one single band in SDS-PAGE indicating that it lacks the extensive phosphorylation found in other isoforms (compare Fig. 2A and B). On the other hand the FLAG-tagged Pl isoform which differed from Ps by 47 N-terminal amino acids migrated as two bands. The difference between the mobility of these bands suggested multiple phosphorylation (designated as Y-phosphorylation).

The shortest isoform (Ss), while lacking N-terminal sequences was still strongly phosphorylated. This phosphorylation pattern was designated as X-phosphorylation (Fig. 2A, B) and was absent in PHF10-S isoforms containing С-terminal-PHD domains. In line with these results the FLAG-tagged Sl isoform that had both N- but lacked C-terminal PHDs migrated as three bands, with both Y- and X-phosphorylation. Dephosphorylation of protein extracts with lambda prosphatase completely abolished phosphorylation of PHF10 isoforms leading to single bands on Western blot, corresponding to each transcript (Fig. 2C and also Brechalov *et al*.^5^). It should be mentioned that the dephosphorylated isoform PHF10-Sl migrates faster than the fastest migrating band in the untreated sample. This suggests that, PHF10-Sl while does not have the extensively phosphorylated sites as other isoforms is still phosphorylated on some minor sites. In summary all PHF10 bands detected in extracts are phosphorylated.

Next, cells were treated with CHX and the time course of protein abundance (stability of isoforms) was studied (Fig. 2D). The transcription level was similar for all four isoforms (Supplement, Fig. S1) but the protein levels of each isoform differed (Fig. 2D, time 0) indicating a differential regulation of their translation or degradation.

We also found that X-phosphorylation increased the stability of PHF10-S isoforms. This can be observed by CHX time course of PHF10-Ss and PHF10-Sl. The lower band corresponding to the protein lacking X-phosphorylation disappeared more rapidly than the upper bands. Comparison of Pl vs Sl and Ps vs Ss isoforms demonstrated that PHF10-P isoforms degraded more rapidly than PHF10-S isoforms having X-phosphorylation (Fig. 2D). In summary, isoforms that are capable of X-phosphorylation show increased stability.

We compared the degradation of PHF10 in nuclei and the cytoplasm. The endogenous PHF10 in nuclear of cytoplasmic extract of HEK293 treated with CHX degraded in course of time both in nucleus and cytoplasm. To differ between the distinct PHF10 isoforms HEK293 cells were transfected with different FLAG-tagged PHF10 proteins treated with CHX for different time and separated into nuclear and cytoplasmic fractions. Our data demonstrate that, both in the nuclei and the cytoplasm PHF10-S were more stable than PHF-P isoforms, and the stability of phosphorylated PHF10-S isoforms was higher than that of non-phosphorylated PHF10-S (Fig. 2E). As the whole FLAG-tagged nuclear PHF10 is associated with BAF200 (Fig. 2F) we conclude in nuclei PHF10 degrades being incorporated in PBAF complex. Overall, data suggest similar mechanisms of PHF10 stabilization and degradation in the nucleus and the cytoplasm.

### PHF10 degradation is targeted by β-TrCP ubiquitin ligase

**Figure 3:**
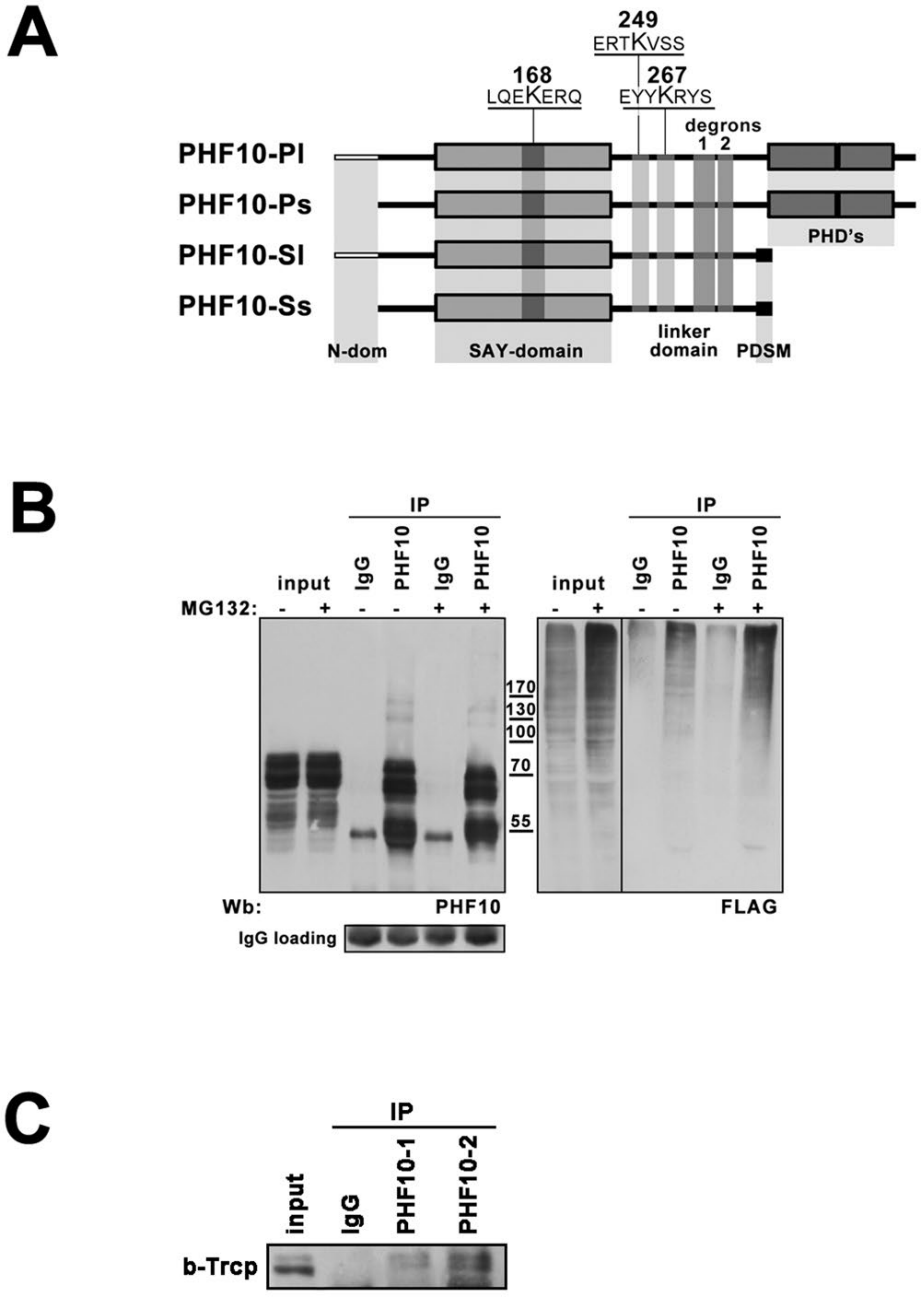
PHF10 is degraded via β-TrCP targeted ubiqutination. (**A**) The schematic representation of localization of ubiqutination sites and β-TrCP degrons within PHF10 isoforms. Sequences containing ubiquitinated lysines (according to Phosphosite database) are underlined. The ubiquitinated lysines and β-TrCP degrons are common for all PHF10 isoforms. (**B**) The HEK293 cells were transfected with FLAG- ubiquitin and treated (if indicated) with 10 µM MG132 for 8 hrs. Then PHF10 was immunoprecipitated from control and treated HEK293 extracts with Protein A –Sepharose bound anti-PHF10 antibodies. IgG coupled to protein A Sepharose was used as a control of unspecific precipitation. Western blot of ubiquitinalated PHF10 was developed with anti-PHF10 (left panel) and anti-FLAG (right panel) antibodies. The antibody against the light chain of IgG was used as secondary antibody. The lower panel demonstrates the same Western blot developed with only secondary antibody to demonstrate that equal amounts of antibodies were used for immunoprecipitation. (**C**) PHF10 and β-TrCP interact in HEK293 extract. Immunoprecipitation of β-TrCP from HEK293 extracts with antibodies against PHF10. IgG coupled to protein A Sepharose was used as a control of unspecific precipitation. The precipitated proteins were resolved by SDS-PAGE and visualized by Western blotting. The images in Fig. 3(B and C) are cropped. The full length of Fig. 3B is presented in Supplementary Material.

To further study PHF10 degradation we identified its potential ubiquitination sites by searching the PhosphoSitePlus repository^21^ which contains phosphoproteomic and ubiquitomic data. We found one ubiquitination site in the SAY-domain (Lys 168) and two sites (Lys 267 and Lys 249) in the linker domain (the numbering of amino acids starts from the first methionine of PHF10-Pl isoform). These sites were shared by all PHF10 isoforms (Fig. 3A).

To discriminate between mono- or polyubiquitination of PHF10 we expressed FLAG-tagged ubuiqitin in HEK293 cells and precipitated the endogenous PHF10 protein with anti-PHF10 antibodies (Fig. 3B, left panel). We did not identify any FLAG-ubuiqitin marked major PHF10 bands corresponding to mono-ubiquitination, but observed a number of minor bands of higher molecular weight when developed the same blot with anti-FLAG antibody (Fig. 3B, right panel). The intensity of these bands significantly increased when the cells were treated with the proteasome inhibitor MG132 to prevent the degradation of polyubiquitinated proteins (Fig. 3B, right panel). These results demonstrated that PHF10 is indeed a target for polyubiquitin-dependent 26S proteasome degradation.

The analysis of PHF10 amino acid sequence revealed two motifs represented by glycine surrounded by acidic amino acids and serine residues that resembled the phosphodegron for β-TrCP ubiquitin ligase (DpSGXX(X)pS), a motif that undergoes diphosphorylation and mediates the interaction of the substrate with WD40 repeats of β-TrCP. The sequences containing potential non-canonical β-TrCP degrons (295-D**S**DGD**S**DDGED-307 and 319-DSSSGNV**S**EGE**S**PPDS-336) were localized in the linker domain shared by all PHF10 isoforms (Fig. 3A).

A number of non-canonical β-TrCP phosphodegron sequences has been reported^22–24^. The β-TrCP degrons identified by FIMO analysis in different proteins^10^ include the canonical and non-canonical β-TrCP degrons. Interestingly, four non-canonical degrons were identified in PHF10^10^. In accordance with the p-values and conservation score, only two of them had a high probability of interaction with β-TrCP (Supplement, Table S1). These degrons were localized in the linker domain and overlapped with degron sequences identified in our study.

In co-immunoprecipitation experiments with antibodies against PHF10 or β-TrCP from HEK293 cell extract (Fig. 3C) the anti-PHF10 antibodies co-precipitated some amount of β-TrCP suggesting their interaction. The observed interaction was not strong due to transient physical association of β-TrCP with its substrates, identified for E3 ubiquitin ligases^25^. This data indicated that PHF10 degradation indeed involves β-TrCP ligase.

### β-TrCP RNAi increases the level of PHF10 and other PBAF subunits

**Figure 4:**
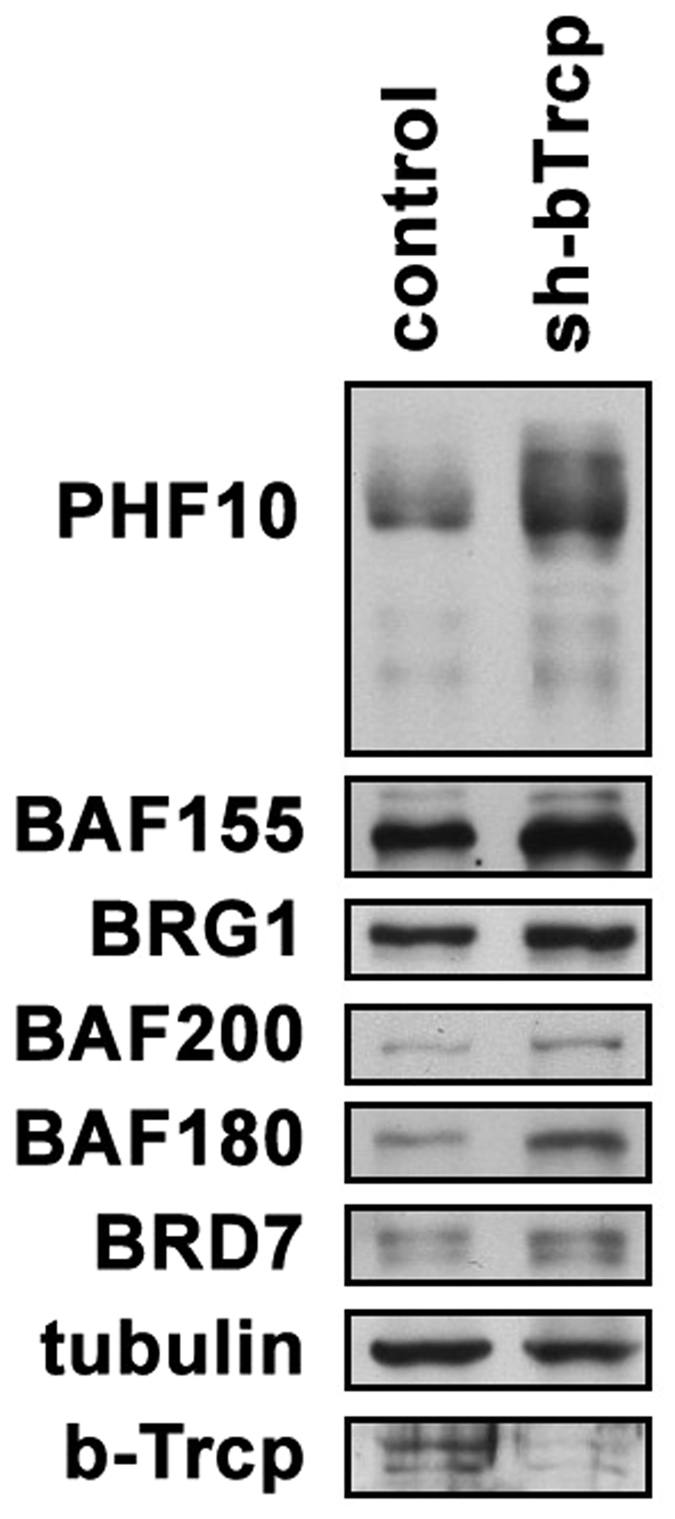
The influence of β-TrCP knockdown on the level of PBAF subunits. (**A**) HEK293 were transfected by sh-β-TrCP-pSuper and control empty vector. Two days later cells were lysed and the level of PBAF subunits was analyzed by Western blot using the indicated antibodies. Tubulin was used as loading control. The lower panel demonstrates the level of β-TrCP knockdown. The image is cropped.

Our data demonstrated that β-TrCP E3 ubiquitin ligase targeted PHF10 to degradation. To provide further evidence for this mechanism we studied the influence of β-TrCP knockdown on the amount of PHF10 and other PBAF subunits, in particular, subunits of the signature module, in the nucleus. The β-TrCP in human cells is represented by two homologues (β-TrCP1 and β-TrCP2) which functionally do not differ from each other. The shRNA knockdown of β-TrCP homologues in HEK293 cells decreased the level of β-TrCP mRNA twofold. Concomitantly, we observed a significant increase of PHF10 protein following β-TrCP knockdown (Fig. 4A).

The knockdown of β-TrCP also led to an increase of several other SWI/SNF subunits including the core (BRG1 and BAF155) and PBAF- specific ones (BAF200, BAF180 and BRD7) (Fig. 4A). As a major amount of PBAF subunits in the nuclei is present in a complex we suggested that stabilization of PHF10 by RNAi of β-TrCP may also stabilize other PBAF components, in particular, the members of its signature module. An alternative variant was that the other PBAF subunits were also degraded by β-TrCP. To address this question we studied whether PBAF subunits contain the potential sites of interaction with β-TrCP using the data from Low *et al*.^10^. Among the PBAF signature module members, besides PHF10, only BAF180 had 4 potential β-TrCP binding sites. Most probably, the increase of BAF200 and BRD7 levels in β-TrCP RNAi cells occurs due to their stabilization by interaction with PHF10 or BAF200 within the PBAF signature module. Interestingly, BAF53B and BAF45D (DPF2), the tissue - specific subunits of SWI/SNF also had one and two β-TrCP binding motifs, respectively.

Among the core PBAF subunits multiple sites for potential interaction with β-TrCP were found in BRG1 ATPase (7 sites) or its homologue BRM (3 sites). One potential site was found in BAF57. No sites were detectable in BAF155 whose level also increased after the knockdown, indicating that BAF155 is stabilized by interaction with BRG1^1^.

### The serine residues within β-TrCP degrons are phosphorylated by CK-1 protein kinase in PHF10-S but not in PHF10-P isoforms

**Figure 5:**
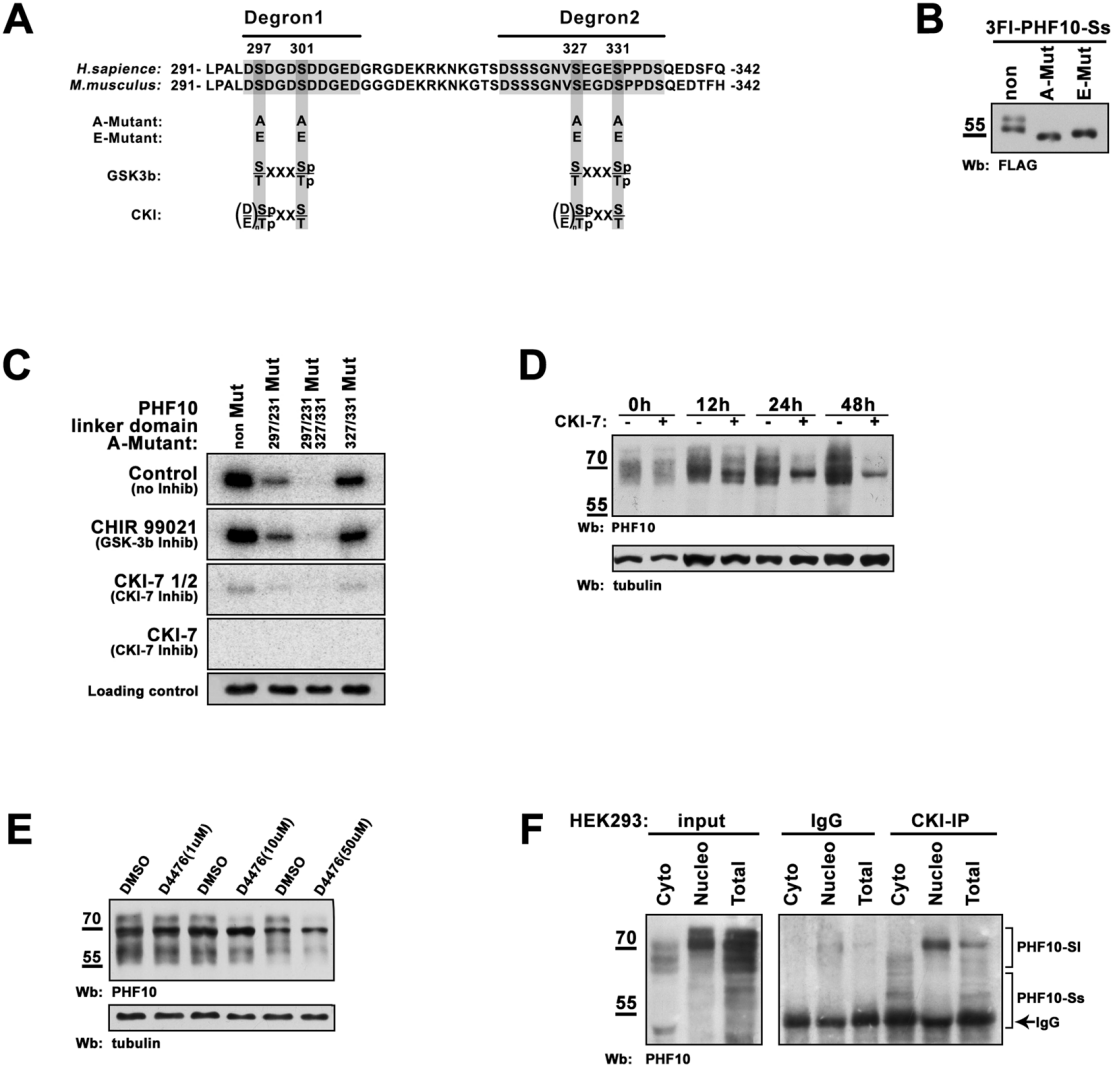
The serines within β-TrCP degrons are phosphorylated by CK-1. (**A**) The amino acid sequence of PHF10 linker domain (amino acids 291–342) which contains β-TrCP degrons (shown in grey boxes). Serines 297, 231 and 327, 331 which undergo phosphorylation are highlighted by dark-grey boxes. They were replaced by alanines (A-mutant) or glutamic acids (E-mutant) in PHF10-Sl isoform. The motifs for GSK-3β and CK-1 phosphorylation are shown below. (**B**) Western blot of FLAG-tagged PHF10-Ss, A-mutant and E-mutant expressed in HEK293 demonstrates that both mutants lack phosphorylation. The numbering of amino acids starts from the first methionine of PHF10-Sl isoform. (**C**) Testing the GSK-3ß and CK-1 for the ability to phosphorylate PHF10-S. The 6His-tag PHF10 linker domain (amino acids 291–342), and its mutated variants were expressed within *Escherichia coli* system, purified and incubated with HEK293 extract supplied with γ-^32^ P-ATP. Extracts of HEK293 without any inhibitors (upper panel), or with GSK-3ß inhibitor CHIR 99021 (the second panel), with 12 µM or 6 µM of CKI-7 inhibitor (the third and the fourth panels) were used for treatment of PHF10 purified recombinant proteins. γ-^32^ P-ATP was added to every probe. Purified and immunostained 6His-2 domain was used as loading control. The lower panel (loading control) demonstrates Western blot of aliquots of the above reaction mixtures stained with antibodies against PHF10 linker domain. (**D**) HEK293 cells were treated by CKI-7 inhibitor for 12, 24 and 48hrs. Equal amount of control and treated extracts were loaded in a SDS-PAGE and analyzed by Western blot. It is clearly seen that PHF10 stability decreases upon treatment with CKI-7 inhibitor. (**E**) HEK293 cells were treated with different concentrations of D4476 inhibitor. Equal amount of control and treated extracts were loaded on a SDS-PAGE and analyzed by Western blot. It is seen that PHF10 stability starts to decrease at concentration 10 uM of D4476. (**F**) PHF10 is co-precipitated with anti-CK-1 antibodies from total, nuclear or cytoplasmic extracts. Incubation of the same extracts withIgG-bound  Protein A-Sepharose was used as control for unspecific precipitation of PHF10. The images in Fig. 5(B,C,D,E,F) are cropped. The full-length image for Fig. 5F is presented in the Supplementary Material.

As interaction of β-TrCP with its targets requires phosphorylation we analyzed the phosphorylation of serines within β-TrCP degrons of PHF10. Search across the phosphosite.org database^21^ revealed three serines (Ser 297, Ser 301 and Ser 327) more frequently phosphorylated in PHF10 (found 26 times in different screenings). All of them were located in the identified β-TrCP degrons (Fig. 5A). In addition, Ser 331 present in degron 2 was also phosphorylated (found 4 times).

We then analyzed if Ser residues 297, 301, 327 and 331 were phosphorylated in HEK293 cells. To this end the four Ser residues in PHF10 were replaced either by alanines (A-mutant) to prevent phosphorylation, or by glutamic acid (E-mutant) which mimics constantly phosphorylated serines (Fig. 5A).

We observed that phosphorylation of Ser 297, 301, 327 and 331 occurred only in PHF10-S isoforms as Ser-Ala mutation completely abolished X-phosporylation (Fig. 5B). The FLAG-tagged PHF10-Ss isoform (which has only X-phosphorylation) expressed in HEK293 cells migrated as two bands in SDS-PAGE, the upper band corresponding to a phosphorylated form. In the same time both PHF10-Ss mutants migrated as single bands corresponding to non-phosphorylated PHF10-Ss (Fig. 5B).

To confirm the phosphorylation of β-TrCP degrons the kinase assay was performed. The wild type linker domain (238–361 amino acids), the same fragment with either Ser-297 and -301 mutated to alanines, or Ser-327 and -331 mutated to alanines, or with all four Ser residues replaced with Ala residues (Fig. 5A) was expressed in *E*.*coli*, purified and incubated with HEK293 lysates with the addition of radioactively labeled γ-^32^ P-ATP (Fig. 5C, upper panel). The mutations 297/301 (lane 2) and 327/331 (lane 4) decreased the radioactive signal, while mutations 297/301/327/331 (lane 3) completely abolished the radioactive signal indicating that serines in both degrons underwent phosphorylation.

Next, we aimed to identify protein kinases that regulate PHF10-S stability. The amino acid sequences where the studied Ser residues were localized (DSDGDSD and SEGES) resembled the target sequence for CK-1 and GSK-3β protein kinases (Fig. 5A). Both enzymes have a broad range of substrates and their action often leads to subsequent degradation of the target protein^26, 27^. To identify if these protein kinases can phosphorylate the serines of PHF10-Ss we performed the kinase assay as described above with the addition of 10 uM CHIR 99021, the inhibitor for GSK-3β or 6 and 12 uM of CKI-7, the inhibitor for all seven types of CK-1 (Fig. 5C, second panel and the panels below). No changes of phosphorylation were detected with CHIR 99021. In contrast, the signal completely disappeared if CKI-7 was added indicating that the X-cluster Ser residues were phosphorylated by CK-1.

To determine how inhibition of casein kinases influences the native PHF10, we treated HEK293 cells with the CKI-7 inhibitor at various time intervals and detected both disappearance of the band corresponding to X-phosphorylated PHF10-S forms and an increased degradation of endogenous PHF10 isoforms after 12 h with CKI-7 (Fig. 5D). To confirm this result we used D4476, the other CK-1 inhibitor, which was shown to be very specific^28^. The time-course indicates that in cells treated with D4476 endogenous PHF10 degrades significantly more rapidly than in wild-type cells (Fig. S2). When cells were treated with different concentrations of D4476 (Fig. 5E) the degradation of PHF10 started to be seen at concentration 10 uM while the normal working concentration of D4476 is 50–100 uM.

Finally, we verified PHF10 and CK-1 interaction in co-immunoprecipitation experiments. Antibodies against CK-1 efficiently specifically co-precipitated PHF10-S isoforms from both cytoplasmic, nuclear or the whole cell extract of HEK293 (Fig. 5G).

### Phosphorylation of serine residues in β-TrCP degrons of PHF10-S isoforms opposes their degradation

**Figure 6:**
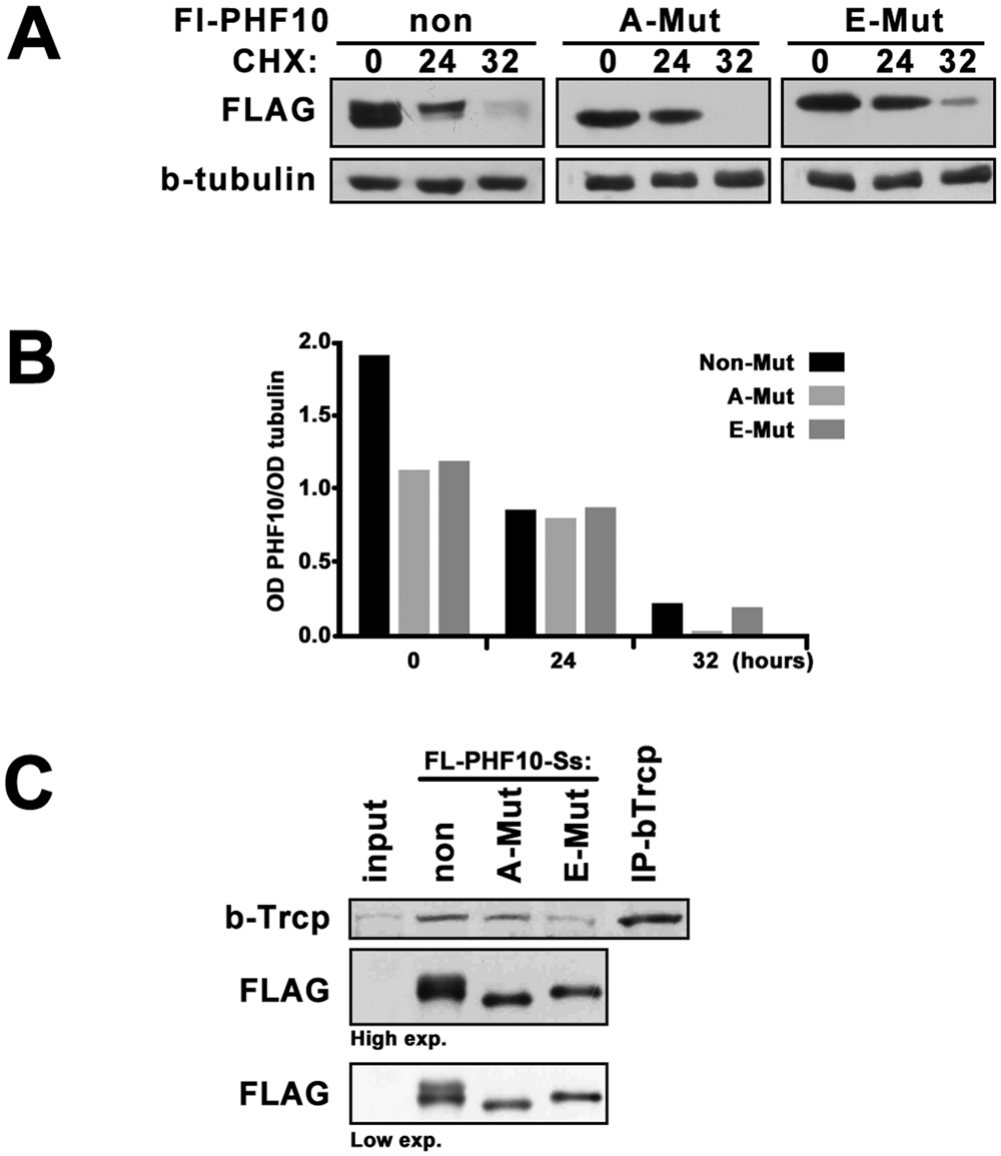
Comparing of the wild-type and mutated PHF10-Ss isoforms. (**A**) HEK293 cells transfected with FLAG-tagged PHF10-Ss, A-mutant or E-mutant were treated with CHX (20 mg/ml) for indicated time. Extracts were analyzed by Western blot, β-tubulin was used as loading control. E-mutant degraded slower than A-mutant similar to the X-phosphorylated (upper band) and non-phosphorylated (lower band) forms of wild-type PHF10-Ss. (**B**) The intensity of bands on Western blot was quantified by densitometry as described in Methods section. (**C**) HEK293 cells were transfected by FLAG-tagged PHF10-Ss, A-mutant or E-mutant isoform and PHF10 forms were precipitated from the extract of transfected cells on FLAG-agarose. Western blot was stained with antibodies against β-TrCP or FLAG. The wild-type PHF10-Ss and A-mutant co-precipitated β-TrCP stronger than E-mutant. Precipitation with anti-β-TrCP antibodies was used as positive control. Note that the level of precipitated FLAG-PHF10 in all lanes was equal (the wild type PHF10 isoform migrates with two bands, the upper one corresponds to phosphorylated form and thus does not interact with β-TrCP). The images in Fig. 6(A and C) are cropped.

Influence of β-TrCP degron phosphorylation on degradation of PHF10-S isoforms was studied. The stability of wild type PHF10-Ss isoform and its A-mutant and E-mutant in HEK293 cells was evaluated using CHX (Fig. 6A,B). Unexpectedly the non-phosphorylated A-mutant degraded more rapidly that the wild type form. Conversely, the E-mutant that mimics constantly phosphorylated Ser residues was even more stable than the wild type protein. These results demonstrated that phosphorylation of serines within the β-TrCP degrons led to stabilization of PHF10-S isoforms (Fig. 6A,B).

As the classical β-TrCP degrons need phosphorylation for efficient interaction with β-TrCP we investigated the interaction of β-TrCP with the wild type and mutated FLAG-tagged PHF10-Ss forms in co-immunoprecipitation experiments (Fig. 6C). Our results showed that while A-mutant co-precipitated β-TrCP similarly to wild type form, β-TrCP interaction with E-mutant was much weaker. Note that the level of precipitated FLAG-PHF10 in all lanes was equal (the wild type PHF10 isoform migrates with two bands, the upper one corresponds to phosphorylated form and thus does not interact with β-TrCP). Thus, phosphorylation of β-TrCP degron within PHF10-S isoforms impairs their association with β-TrCP.

Overall, these results demonstrated that while all PHF10 isoforms have β-TrCP degron the PHF10-S isoforms are protected against β-TrCP by phosphorylation of serines within the degrons. This explains our observations that PHF10-S isoforms were more stable in the cells than PHF10-P and that the PHF10-S isoforms become stable when phosphorylated (Fig. 2D).

### Analysis of β-TrCP – PHF10 interactions

As known for the substrates of β-TrCP, the phosphorylation of serines within the degron is required for efficient binding and subsequent degradation by β-TrCP. To study possible differences in the binding of peptides according to their modifications, targeted molecular docking was performed to find the best conformations of β-TrCP – peptide complexes (see peptides in Materials and Methods). Ten conformations for each docked peptide were detectable on the surface of the β-TrCP binding site. At the first step 24 unique peptide conformations located similarly to the known complex (PDBid 1P22) were selected for analysis, since such an arrangement leads to an effective ubiquitination of phospho-β-catenin^13^. At the next step, we calculated binding energy values using Sybyl8 (Table S2). In addition to the calculated binding energy and Vina DockScore, RMSD variations from the reference peptide structure (PDBid 1P22) are also shown in this table.

Since the studied peptides represented only a part of the flexible loop of PHF10 protein, we considered the possibility of extension of these peptides and the localization of the N- and C-terminus while evaluating the docked conformations. Thus, a large number of obtained conformations had no opportunity for further extension due to steric conflicts. Some conformations were placed in the opposite direction compared to the reference β-catenin structure. These results are given in two very right columns in Table S2, where suitable conformations are marked “+”.

**Figure 7:**
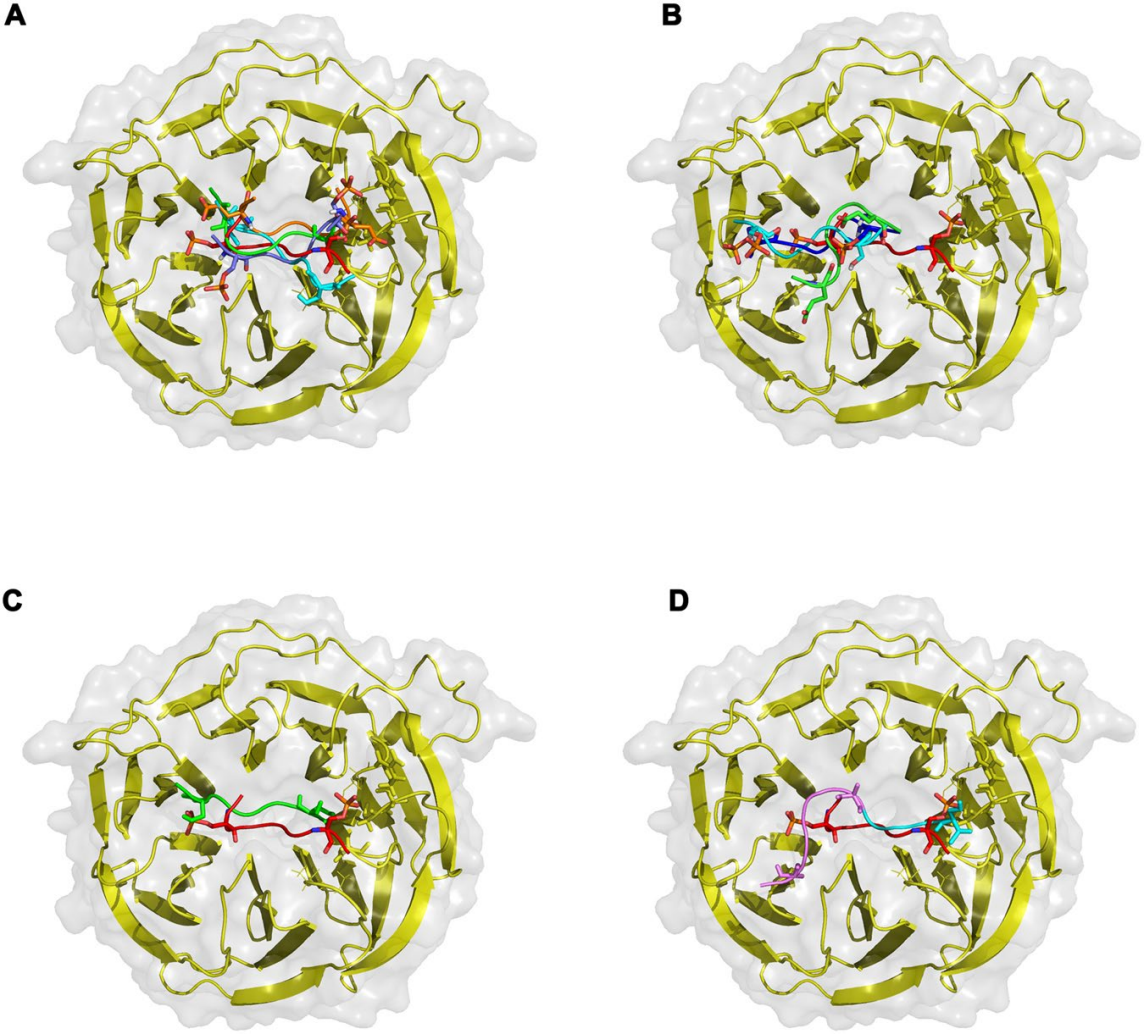
The interaction of PHF10 phosphodegrons with β-TrCP. (**A**) Selected conformations of Peptide-1 variants. Reference β-catenin peptide (PDBid 1P22) is colored red; unphosphorylated variant (Peptide1-Normal) - light blue, Ser-Ala variant (Peptide1-A) - green; phosphorylated variant (Peptide1-P) – violet, Ser-Gln mutated variant (Peptide1-E) – orange. Difference between the reference β-catenin peptide (red) position on the surface of the β-TrCP enzyme and selected conformations of (**B**) single phosphorylated (Peptide2-P1, light blue), double-phosphorylated (Peptide2-P2, blue) and Ser-Gln (Peptide2-E, green) variants of peptide-2; (**C**) unphosphorylated peptide-2 variant (Peptide2-Normal, green); (**D**) Ser-Ala mutated variant (Peptide2-A, violet), C- terminus sequence PPDS, containing Ser16 residue is highlighted cyan.

Studying Peptide-1 and its variations we found that the best conformations were obtained for the non-phosphorylated form (Supplement. Table S2, Peptide1-Normal, model6) and for one Ser-Ala mutant (Table S2, Peptide1-A, model3). On the other hand the phosphorylated (Table S2, Peptide1-P, model2) and Ser-Gln mutated (Table S2, Peptide1-E, model7) peptide-1 forms bound to β-TrCP with much lower affinity than the non-phosphorylated proteins. The binding energy values for phosphorylated/non-phosphorylated peptide-1 were −74.29 and −169.13 kcal/mol, respectively. Likewise, Peptide-1-E-variant had ~3 times lower affinity to WD-domain of β-TrCP than Peptide1-A-variant (calculated binding energies −92,63 and 251,32 kcal/mol, respectively). Best Peptide-1**–**β-TrCP complexes are shown in Fig. 7А.

Evaluation of docking results of Peptide-2 variants we found that 1Ser- and 2Ser- phosphorylated peptides had one of the best binding energies (Table S2, Peptide2-P1, model1; Peptide2-P2, model4) with the respected values −82.91 and −119.72 Kcal/mol. The Peptide-2-E variant had one of the worst affinities (calculated binding energy −39.41 Kcal/mol). Further visual structure analysis showed that the location of Peptide-2-P1, Peptide-2-P2 and Peptide-2-E on the surface of the binding site significantly differed from the reference β-catenin peptide (Fig. 7B–D). Such conformational shift of phosphorylated and Ser/Gln-mutated variants might be a reason for a lack of further ubiquitination of the PHF10-loop, despite the good values of the binding energy (in case of phosphorylated variants).

At the same time, the best conformations of unphosphorylated and Ser/Ala-mutated variants of Peptide-2 had (VinaScore, Sybyl ΔG) no great affinities to WD-domain of β-TrCP but were situated much closer to the reference β-catenin. Their calculated binding energies were −38.58 kcal/mol (Pept2-Normal, model 7) and −87.21 kcal/mol (Pept2-A, model 2). Figure 7C shows the best unphosphorylated peptide-2 variant, and Ser-Ala mutant is represented at Fig. 7C.

In summary, our docking procedures revealed that Peptide-1 had better affinity to WD-domain of β-TrCP enzyme than unphosphorylated and Ser-Ala mutant, which correlates with our experimental data. The phosphorylated and Ser-Gln mutated variants of Peptide-2 can bind to β-TrCP but there location on the surface of WD-domain dramatically differed from the reference β-catenin peptide that could result in the absence of further ubiquitination. The non-phosphorylated peptide-2 variants bind to the enzyme similarly to the reference from 1P22, although with a poor affinity. The Ser-Ala variant of peptide-2 has a spatial shift while it binds to β-TrCP-1.

## Discussion

We show that the PHF10 subunit of the PBAF signature module is the most unstable PBAF subunit. Our study demonstrates an important role of β-TrCP ubiquitin ligase in regulation of PHF10 level and PBAF in general. We found that PHF10 isoforms contained two non-canonical β-TrCP degrons and PHF10 interacted with β-TrCP in co-immunoprecipitation experiments. Interestingly, individual PHF10 isoforms are differentially sensitive to β-TrCP-mediated dergadation as phosphorylation of β-TrCP degrons by CK-1 in PHF10-S isoforms affected their association with β-TrCP and increased their stability. The knockdown of β-TrCP strongly stabilized PHF10 as well as the signature module of PBAF including the subunits that do not contain β-TrCP degrons. The RNAi of β-TrCP also stabilized the core PBAF subunits such as BRG1 ATPase or BAF155 indicating that β-TrCP may participate in the regulation of the entire PBAF complex. Prevention of degradation of β–TrCP-dependent subunits can stabilize other subunits and the PBAF complex.

The β-TrCP ubiquitin ligase is a powerful regulator of protein degradation. This enzyme targets to degradation such important molecules as IκB, β-catenin, and SNAIL^14, 29, 30^ and plays a role in controlling cell cycle progression and carcinogenesis^31, 32^. The β-TrCP has also emerged as a key player in the S and G2 phase DNA damage response checkpoints^33, 34^. Here we showed the novel function of β-TrCP in regulating the PHF10 subunit of the PBAF signature module. Interestingly PBAF is also involved in the control of cell cycle^35, 36^ and PHF10-Pl subunit was shown to activate cell cycle traverse^5^.

Our data demonstrates that PHF10 is the most unstable subunit of the PBAF complex. Thus, rapid degradation highlights the importance of the protein for regulation of gene expression, so that its level in the cell must be strictly controlled. It is also possible that the rate of PHF10 degradation is related to processes that require periodical changes in protein concentration, such as cyclins in the cell cycle, initiation of proliferation (c-Myc) or response to DNA damage (p53). Previously we and others had shown that PHF10 is important for neural differentiation, control of proliferation and apoptosis, but the mechanism whereby PHF10 functions in these events remains poorly understood. What role can PHF10 play in PBAF regulation? Earlier we have demonstrated that PHF10 depletion by RNAi in mammalian cells destabilizes the subunits of the signature module decreasing their abundance in the cells^5^. The same was shown in *Drosophila* ^6^. In the present study we demonstrated that β-TrCP RNAi stabilized PHF10 and other subunits of the signature module. So one may suggest that β-TrCP targeted PHF10 degradation is a trigger for elimination of the PBAF signature module. The signature module was shown to be essential for binding of PBAF to target sites on chromatin^3^. Perhaps the destabilization of the signature module affects the association of PBAF with its cognate DNA binding site. In line with this the knockdown of PHF10 strongly decreased the amounts of other PBAF subunits on gene promoter in *Drosophila* ^6^. Previously we have demonstrated that PHF10-S and PHF10-P isoforms are the subunits of different PBAF complexes^5^. As the stability of PHF10-P and PHF10-S differ the PBAF complexes containing these isoforms may also have different stability.

Interestingly, several other components of the signature module have the predicted β-TrCP degrons^21^. These are the BAF180 (polybromo) subunit (4 degrons) which, similarly to the PHF10 isoform, has chromatin interacting domains: two PHD domains in PHF10-P and seven bromodomains in BAF180. There are also tissue - specific subunits: BAF53B (one degron) and BAF45D (DPF2) (two degrons). The potential β-TrCP degrons were found only in few core subunits: BRG1 (7 degrons), its paralog BRM (three degrons) and BAF57 (one degron). One may suggest that β-TrCP targeted degradation of PHF10 isoforms and other signature module subunits is important for their rapid substitution in the complex and therefore to fine tuning of gene expression.

To allow the binding of β-TrCP the serine residues in a DSGXXS destruction motif need to be phosphorylated by specific kinases. This was demonstrated for canonical as well as for non-canonical β-TrCP degrons^12–14^. However, we for the first time demonstrate that β-TrCP binds to non-phosphorylated destruction motifs within PHF10 and Ser phosphorylation opposes this binding. How this effect may be explained? The PHF10 contains a sequence DSDGDS homologous to the consensus phosphodegron recognized by β-TrCP (DSGXXS), and is required for ubiquitination and degradation. The Ser residue in the DSG stretch of the consensus sequence must be phosphorylated (as shown for CDC25a,b, β-catenin and others). PHF10 is an exception in which the protein becomes more stable, as the degron-like sequence becomes phosphorylated by CK-1. Our computational analysis confirms this surprising finding showing that the first of the proposed degrons has a better affinity to WD-domain of β-TrCP enzyme than unphosphorylated and Ser-Ala mutants, which correlates with our experimental data. While the second degron had better affinity, to the β-TrCP, when the peptide was phosphorylated, such binding required an orientation of the peptide dramatically different from known structures such as β-catenin, while the non-phosphorylated peptides were bound in a canonical manner. This unorthodox mechanism points to a possibility that CK-1 and β-TrCP could not only degrade a set of proteins but also stabilize a different set of targets, the latter acting in an opposite way than the degraded proteins^37^.

Phosphorylation must play an important role in PBAF regulation. A number of PBAF subunits are phosphorylated, and these modifications are required, in particular, for binding to their protein partners^18, 38, 39^. Phosphorylation of main proteins in SWI/SNF can also affect the stability of the complex, as phosphorylation of BRG1 and hBRM led to their dissociation from chromatin in mitosis^40^. However, for many of such modifications no functions are known. For example, BAF155, BAF170 and BAF47 are phosphorylated by Akt protein kinase but the essence of this phenomenon remains to be elucidated^41^.

**Figure 8:**
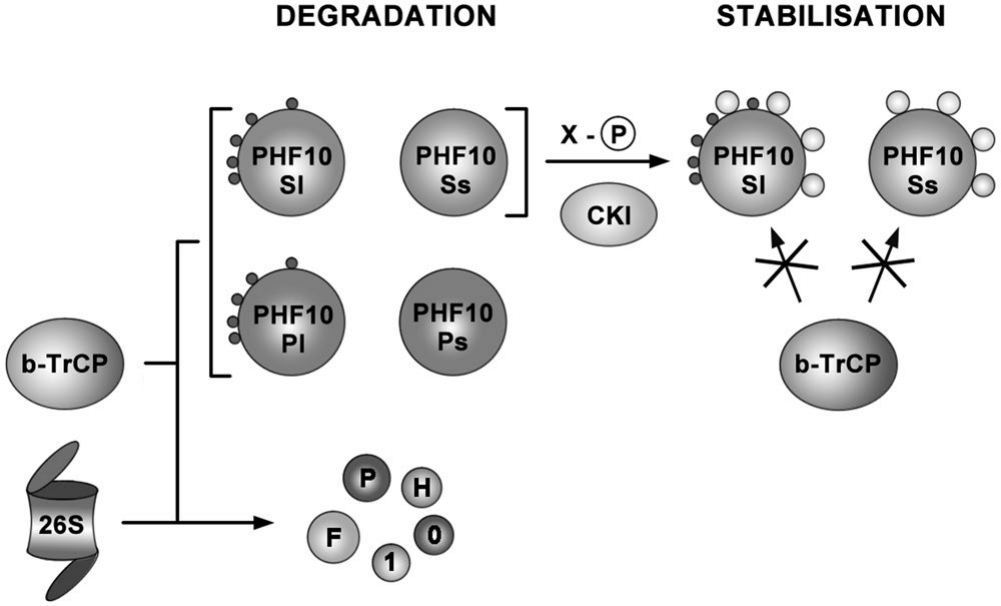
The diagram of PHF10 degradation. PHF10 in mammalian cells is represented by four isoforms which differ by their N-terminal and C- terminal domains. Sl and Pl isoforms have additional 47 N-terminal amino acids while Pl and Ps isoforms have C-terminal double PHD domains. All PHF10 isoforms undergo β-TrCP targeted degradation, however their stability is different and depends upon post-translational phosphorylation which in turn depends upon their domain structure. Two main phosphorylation patterns designated as Y-phosphorylation and X-phosphorylation were identified in PHF10. Y-phosphorylation (black circles) was found in Sl and Pl isoforms and depends upon the presence of 47 N-terminal amino acids. X-phosphorylation (light circles) which is determined by the absence of PHD domains is present in Sl and Ss isoforms. Importantly X-phosphorylation impairs interaction of Sl and Ss isoforms with β-TrCP and thus opposes their degradation.

The phosphorylation of β-TrCP degrons within PHF10 (assigned as X-phosphorylation) depends upon the absence of C-terminal PHD domains, so only PHF10-S isoforms undergo this phosphorylation by CK-1. As a result these isoforms are more stable in the cell. Probably differential stability of isoforms is important for their exchange within the PBAF complex. In such a scenario phosphorylation by CK-1 would lead to stabilization of S-isoforms, while P-isoforms would rapidly degrade (Fig. 8). It is tempting to suggest that different and a complicated regulation of PHF10 isoform stability as well as its rapid degradation is one of the mechanisms of fine regulation of the PBAF complex and gene expression in human cells. Different isoforms interact with different partners through β-TrCP and other mechanisms to achieve optimal stability, thereby allowing the cell to rapidly shift from one type of PBAF complexes to another. Because PHF10 is the only subunit of PBAF with such a rapid turnover while the stable levels of other proteins are strictly controlled^1^ this points to central role of this subunit in the discovered regulatory mechanism.

## Materials and Methods

### Total cell lysates, inhibitors treatment, nuclear and cytoplasmic fractionation

HEK293 cells were grown in DMEM medium supplemented 10% FBS (HyClone), 2 mM L-glutamin at 37 °C, 5% CO2. The cells were treated by CHX 20mkM or 6 and 12 uM (as indicated) of CKI-7 (Sigma). HEK293 cells were lysed in Lysis Buffer: 10 mM HEPES (pH 7.9) containing 5 mM MgCl, 0.5% Nonidet P-40, 0.45 M NaCl, 1 mM DTT, a protease inhibitor cocktail (PIC) (Roche) and a 1% Phosphatase inhibitor cocktail 3 (PhIC) (Sigma-Aldrich). The lysate was centrifuged at 10 000 rpm, 4 °C, for 10 min, and the supernatant was diluted 4-fold with the same buffer but without NaCl. The extract was treated with DNAse I (USB, 0.6 units/mL) and RNAse (Stratagene, 10 units/mL).

For cellular fractionation cells were lysed in FLB buffer (40 mM Tris-HCl, pH = 7,8; 100 mM NaCl; 2,5 mM MgCl2, 1 mM DTT; PIC; PhIC) on ice, grinded in Loose Dounce, centrifuged for 1 minute and supernatant was employed as the cytoplasmic fraction. The pellet was resuspended in Lysis Buffer, grinded in Thight Dounce, incubated 10 minutes on ice, centrifuged as mentioned above and diluted the same way.

The probes were equalized using Qubit Protein Assay Kit (ThermoFisher Scientific), mixed with 4X LB (200 mM Tris-HCl, pH = 6.8; 4% SDS; 40% Glycerol; ~0,01% Bromphenol blue; 100 mM DTT), and boiled for 10′.

Quantification of the Western blots was done using ImageJ gel analysis software (NIH, USA)^42^.

### Antibodies and immunoprecipitations

Polyclonal antibodies were generated by immunizing rabbits with the following His-tagged polypeptides: amino acids 29–266 and 259–536 of β-Trcp; amino acids 5–220 of cMyc; or GST-tagged 216–296 BRG1 (H88). Animal handling was done with ethics approval from Engelhardt Institute of Molecular Biology, Russian Academy of Sciences and in accordance to local regulations and guidelines. All antibodies were affinity purified. Antibodies against PHF10, BAF200, BAF155, BRD7, BAF180 were developed in our lab earlier and described in ref. 5. Antibody to FLAG (M2 monoclone) was from Sigma-Aldrich; to β-tubulin (clone E7) from DSHB; to actin (clone 13E5) from Cell Signaling; to CK-1 (#2655) from Cell Signaling.

Immunoprecipitation was performed by adding 15 μL of antibody-saturated protein A Sepharose beads (Sigma-Aldrich) to the cell extracts (2 × 10^6^ cells per round) and incubating the mixture overnight at 4 °C on a shaker. The beads were washed 3 times with 1 × PBS containing 0.5 M NaCl and 0.1% Triton X-100, and the bound proteins were eluted with 2 bead volumes of 1 × PBS with 0.5% N-lauroylsarcosine. Immunoprecipitations of overexpressed FLAG-tagged wild-type PHF10-Ss and A-, E- mutants with FLAG-M2 agarose (Sigma-Aldrich) were performed under the same conditions.

### Plasmids and transfection

For expression of FLAG-fused PHF10 isoforms the coding sequences of transcripts, raised earlier NM_133325.2 (PHF10-Pl), KC839988 (PHF10-Ps), KC839989 (PHF10-Sl), and KC839990 (PHF10-Ss) were cloned into the pcDNA vector. Mutation of 297, 231 and 327, 331 serines into alanins and into glutamic acids were performed with primers: A-Mutant 5′-ctagacgctgatggtgatgcagatgatggc and 5′-gcaatgtagctgaaggggaagcccctcctgac; E-Mutant 5′-ctctagacgaggatggtgatgaagatgatggc and 5′-gcaatgtagaagaaggggaagagcctcctgac. Activated Gly-Gly-C-end form of Ubiquitin was cloned into FLAG-pcDNA vector.

For β-TrCP 1 and 2 simultaneous knockdown the sequence 5′-gtggaatttgtggaacatc was cloned in pSuper vector for generating of short hairpin.

For overexpression experiments HEK293 (transfected with polyethylenimine (PEI) (Sigma-Aldrich)) cells were harvested 3 days after transfection with a change in culture medium 24 h after transfection.

### *In vitro* kinase assay

For *in vitro* kinase assay linker domain (238–361 aa) and A-Mutant forms were clone in pET22b vector and then recombinant 6His-Linker Domain proteins expressed in *E. coli* and purified at Ni Sepharose (GE Healthcare). For the *in vitro* kinase experiments, 1 ug 6His-Linker Domain or the same ammount of mutants forms were incubated with 100 ul of HEK293 extracts or extracts with inhibitors of 12 uM CK-1 (CKI-7, Sigma-Aldrich) or GSK-3ß (CHIR 99021, Sigma-Aldrich) in the presence of γ-^32^ [P]-labeled ATP at 37 °C. Than the samples were processed for autoradiography or western blot as specified.

### Molecular docking

The spatial structure of β-TrCP1 WD-domain was taken from the bank of three-dimensional structures PDB (PDBid - 1P22)^13^. Initial optimization and preparation of the receptor structure was carried out using Sybyl8.1 (Tripos L.P.) and AutodockTools^43^ software. Using Sybyl8.1 we constructed the following peptides:

Peptide1-Normal - D**S**DGD**S**DDGED

Peptide1-A- D**A**DGD**A**DDGED

Peptide1-E - D**E**DGD**E**DDGED

Peptide1-P- D**S**(-**p**)DGD**S**(-**p**)DDGED (all highlighted serines are phosphorylated)

Peptide2-Normal - DSSSGNV**S**EGE**S**PPDS

Peptide2-A - DSSSGNV**A**EGE**A**PPDS

Peptide2-E - DSSSGNV**E**EGE**E**PPDS

Peptide2-P1 - DSSSGNV**S**(-**p**)EGE**S**PPDS (only the first highlighted serine is phosphorylated)

Peptide2-P2 - DSSSGNV**S**(-**p**)EGE**S**(-**p**)PPDS (all highlighted serines are phosphorylated)

As we studied only small parts of the PHF10 protein flexible loop, CH3- caps were placed at the N- and C- terminus of the constructed peptides to eliminate unwanted charges at the ends, that could affect docking results. Docking was performed using Autodock Vina^43^. During molecular docking the receptor molecule remained rigid while the peptides were treated as flexible. All of the obtained peptides conformations were evaluated based on Vina DockScore, RMSD calculations and the interaction energy with the help of Sybyl8.1 software. The binding energy calculations were performed based on the individual free energy values of peptides, receptor molecule (TrCP1) and their complexes. During free energy calculations Tripos force field was used for energy minimization procedures. Partial charges were calculated using the Gasteiger-Huckel method. The final values of binding energies were calculated using the equation:

$${{\rm{\Delta }}{\rm{G}}}_{{\rm{bind}}}{{\rm{\Delta }}{\rm{G}}}_{{\rm{complex}}}-({{\rm{\Delta }}{\rm{G}}}_{{\rm{receptor}}}+{{\rm{\Delta }}{\rm{G}}}_{{\rm{peptide}}}).$$

## Electronic supplementary material

Supplementary Information
