# Supplementary material for: Stability of the PHF10 subunit of PBAF signature module is regulated by phosphorylation: role of β-TrCP
Source: Sci Rep. 2017 Jul 17;7:5645. doi: 10.1038/s41598-017-05944-3 (PMC5514133; doi:10.1038/s41598-017-05944-3)
Supplement: Supplementary file 1 — Supplementary Information [file 41598_2017_5944_MOESM1_ESM.pdf]

SUPPLEMENT INFORMATION

Stability of the PHF10 subunit of PBAF signature module is regulated by phosphorylation: role of β-TrCP

Victor V. Tatarskiy Jr[3*], Yuriy P. Simonov[2*], Dmitriy S. Shcherbinin[4], Alexander V. Brechalov[2], Sofia G. Georgieva[1,2†], Nataliya V. Soshnikova[1†]

Affiliation:

1- Department of Eukariotic Transcription Factors,

Institute of Gene Biology, Russian Academy of Sciences,

Vavilov Str. 34/5, Moscow, 119991 Russia

2- Department of Transcription Factors,

Engelhardt Institute of Molecular Biology, Russian Academy of Sciences,

Vavilov Str. 32, Moscow, 119991 Russia

3- Laboratory of Tumor Cell Death,

N.N. Blokhin Russian Cancer Research Center

Kashirskoye Shosse 24, Moscow, 115478, Russia

4- Laboratory of Structure Bioinformatics,

Institute of Biomedical Chemistry (IBMC),

Pogodinskaya street 10 building 8, Moscow, 119121 Russia

*- These authors contributed equally to the work

†- Correspondent authors: Dr. Soshnikova Nataliya, so2615nat@gmail.com

Dr. Georgieva Sofia, sonjag@molbiol.edu.ru

Figure S1. The level of mRNAs of FLAG-tagged PHF10 isoforms in transfected HEK293 cells

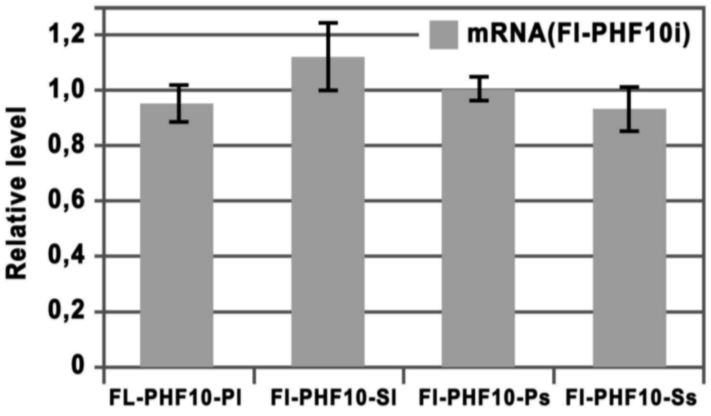

Figure S2. HEK293 cells were treated by CKI-7 inhibitor for 12, 24 and 48hrs. Equal amount of control and treated extracts were loaded in a PAAG gel and analyzed by Western-blot. It is clearly seen that PHF10 stability decreases upon treatment with CKI-7 inhibitor.

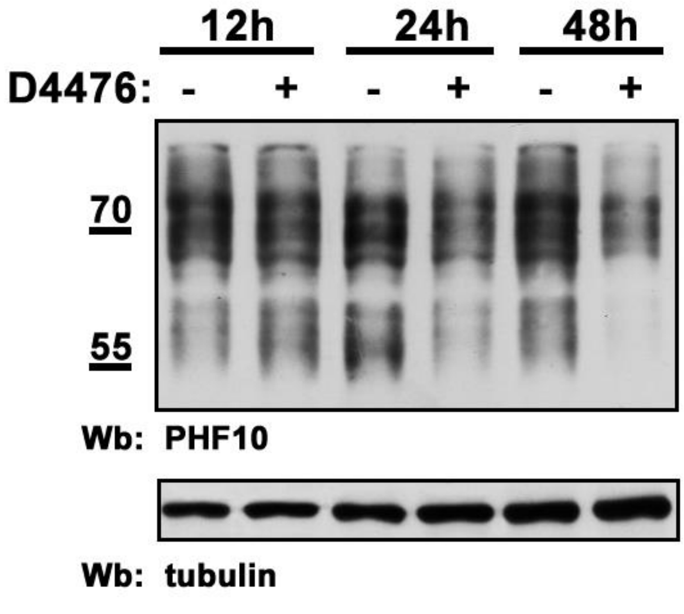

Figure S3. The full-length blot of Figure 3B

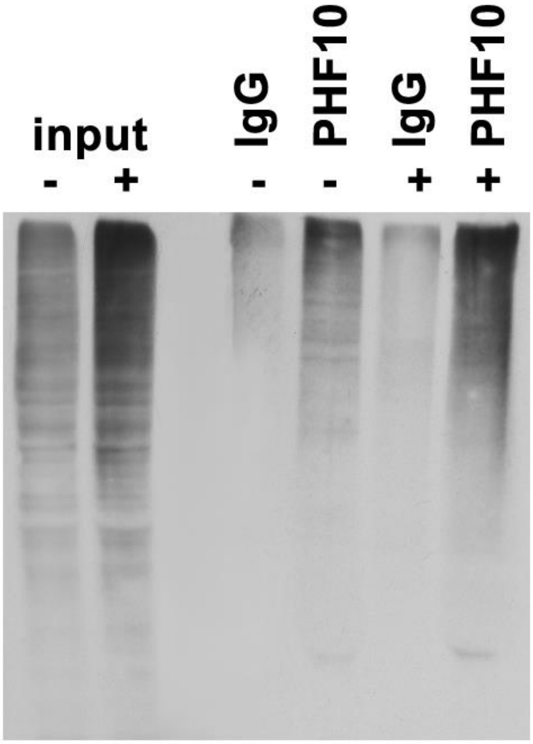

Figure S4. The full-length blot of Figure 5E

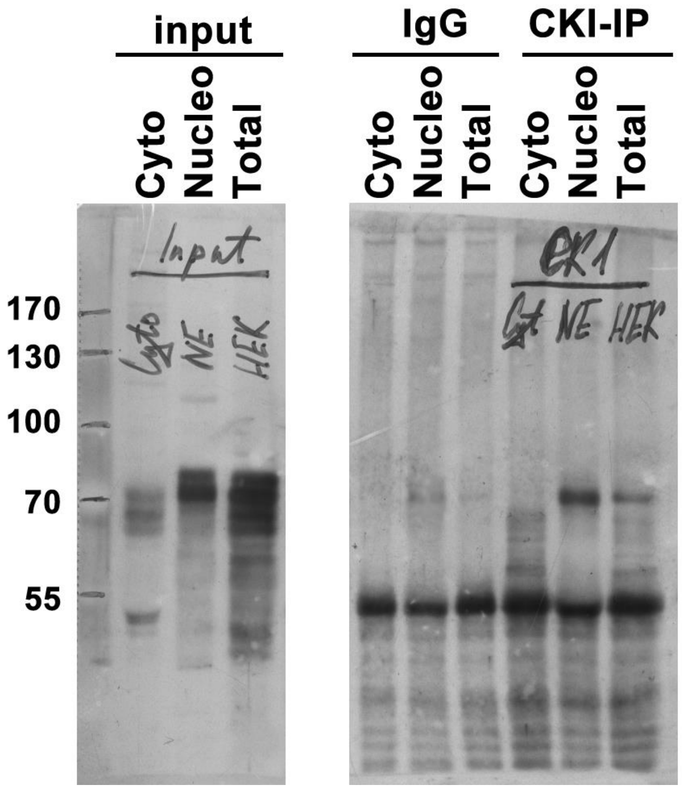

Table S1. Degrones predicted by FIMO analysis, adapted from [11]

RMSD- Root mean square deviation from the reference β-Catenin peptide

Vina Score – Dock score function values

Sybyl ΔG – Calculated binding energy values.

Extension – Possibility of further loop extension

N,C- position – N,C-terminus direction in comparison with the reference β-Catenin structure

Table S1

Ranking In FIMO (P-Value)	Motif Matched Human Sequence	Motif Start Position	Motif Stop Position	FIMO P-Value	Motif Position	Sequence Similarity Between Human Mouse	Sequence Similarity Between Human Fish	Sequence Similarity Between Human Fly
167	SDSSSGNVSE	319	328	2,81E-06	328	1	0,8	0,2
2814	DDGEDGRGDE	302	311	1,06E-04	311	0,9	0,5	0,1
8852	SCETSSQDLG	56	65	4,27E-04	65	0	0,6	0
13529	QCENSGHPSC	403	412	7,14E-04	412	0,9	0,9	0,5

Table S2. Analysis of the best peptides conformations

	RMSD	Vina	SYBYL	Extension	N,C-position
**Peptide1**					
**P, model2**	**9,128**	**-5,8**	**-74,288**		**+**
P, model3	5,874	-5,7	22,656	+	
A, model2	9,405	-6,5	-136,402	+	
**A, model3**	**3,965**	**-6,5**	**-251,53**	**+**	**+**
A, model4	11,738	-6,5	-185,76	+-	
E, model3	8,658	-5,5	-32,764	+	
E, model4	8,428	-5,4	-92,636		
E, model5	9,19	-5,3	53,576	+	
**E, model7**	**8,428**	**-5,3**	**-92,636**	**+**	**+**
Normal, model3	9,053	-6,4	-229,03	+	
**Normal, model6**	**5,171**	**-6,2**	**-169,13**	**+**	**+**
**Peptide2**					
A, model1	8,42	-6,6	-49,722	+	

**A, model2**	**8,067**	**-6,4**	**-87,21**	**+**	**+**
**E, model1**	**5,986**	**-5,8**	**-39,414**	+	+
E, model2	6,602	-5,7	-27,972	+	+
E, model3	6,981	-5,7	-25,036	+	+
Normal, model1	10,639	-6,3	-68,832	+	
Normal, model2	11,457	-6,2	-55,622		
**Normal, model7**	**3,52**		**-38,58**	**+**	**+**
**P1, model1**	**7,219**	**-6,2**	**-119,72**	**+**	**+**
P1, model4	5,955	-6,2	-96,826	+	+
P2, model2	9,849	-5,7	-60,814		
**P2, model4**	**5,982**	**-5,4**	**-82,908**	**+**	**+**
P2, model5	9,678	-5,4	-110,79		
